# Observations on the Qualifications Necessary to a Practitioner of Dental Surgery—and on the Manner in Which They Should Be Obtained

**Published:** 1839-09

**Authors:** Chapin A. Harris

**Affiliations:** Dentist, Baltimore.


### OBSERVATIONS

### *On the qualifications necessary to a Practitioner of Dental Surgery—and on the manner in which they should be obtained.*

---

[ BY CHAPIN A. HARRIS, M. D., DENTIST, BALTIMORE. ]

---

Conducive as is the art of the dentist, when judiciously and skilfully exercised, to the comfort and well-being of man, and when injudiciously and unskilfully, often to his great, and sometimes irreparable injury, one would suppose that it would be entrusted to such only, as were qualified to exercise its duties in a proper manner. This however, is not the case. All who are disposed to occupy themselves with it, are permitted to do so, without any reference to competency whatever ; and to this, the reproach that has been so often visited upon the profession, is mainly to be attributed.

A thorough knowledge of the science of dentistry, is more difficult to be acquired, than is generally supposed. Many regard it as a mere mechanical occupation, and believe that any one possessed of a slight degree of tact and manual dexterity can practice it. But this is a mistaken view of the subject. Dentistry, as it exists at the present day, is a science, partaking of medicine, surgery, and the manual arts ; and he who practices it, should have some knowledge of all these branches, and moreover, be possessed of a mind that naturally inclines him to their study.

So far as the *prosthesis* of the teeth alone, is concerned, a man may be expert and even skilful ; he may be able to supply the loss of a single organ, and so on up to an entire *denture,* upon the most correct principles of dental science, and still be unable to exercise all the duties that pro perly belong to the dentist. In short, he may be a profound mechanician, and thoroughly versed in that branch which relates to *la prothese des organes buccoux,* as the French denominate it, and yet be deficient in many other qualifications that are essential to a skilful practitioner of dental surgery. And equally do the medical and surgical sciences fail to furnish all the information necessary to be possessed upon the subject; and here I would take occasion to remark, that of the graduates in medicine, who have turned their attention to this branch of the healing art, many have shown themselves entirely incompetent to the successful discharge of its duties. In proof of the truth of this assertion, it is only necessary to observe, that men are occasionally to be found, even among the lowest grade of dental practitioners, bearing the dignified title of M. D., and of these, some have had the arrogant audacity to claim, on account of their medical knowledge, a superiority of skill over many of our most distinguished dentists.

That a man, who, after having received a medical education, should adopt, and even descend to charlatanism in the dental profession, as some have done, and thus bring upon both it and themselves obloquy and disgrace, is greatly to be regretted. Charlatanism is bad enough in men, having no such passport to public favor and confidence; but when it is practiced under the sanction of a medical diploma, the enormity of the crime is ten fold greater, and that it has been, and is, even at the present day, many examples might be alleged. We forbear, however, to dwell upon a subject, that must be both painful and humiliating to every honorable, high-minded member of the profession.—Quacks are to be found in all professions,—the medical is far from being exempt from them. Hundreds of examples might be adduced, but we will only mention one, and this was in the person of a foreigner, who, some two or three years ago, visited many of the principal cities and towns in this country, in the character of an Oculist, professing to perform the most wonderful cures. So artfully did he manage, that persons flocked to him from far and near; but his reputation, like that of the Crawcours, of *Royal Mineral Succedaneum notoriety,* was not destined to be of long duration; for the light, of which he deprived his dupes, so opened the eyes of the good people of the United States, that he was soon compelled to seek another field for the practice of his abominable villanies.

So numerous have been the instances of empiricism in medicine, that few countries, at the present day, permit it to be practiced, except by individuals who have been specially educated for the business; nor even then, unless they shall have evidenced that they were qualified to a board appointed for the purpose of examining and licensing candidates for this most responsible and highly honorable calling. People generally, seem to require this, and are unwilling to admit to their confidence, any except those who can exhibit some such evidence of qualification; and well would it be were they equally cautious in reference to the employing of gentlemen of the dental prefession.

But to return from this digression.—Although in a dental education, the general principles of medicine and surgery, should be incorporated, it by no means follows, that in order to be a skilful practitioner of dentistry, it is requisite to graduate in these sciences. The annals of dentistry furnish numerous proofs to the contrary. What names, I would ask, ever stood higher in the profession than Hudson, Gardette, Hayden, Greenwood, Parmly, Baker, Newton, Koecker, Harrington, Waite and Cartwright ?* These gentlemen have long been identified with the profession, Dr. Hayden for upwards of forty years, and have enjoyed an almost unexampled reputation for skill and professional acumen. We might mention the names of many others, who, if they have not been as long known in the profession, have acquired for themselves, a reputation that will vie with that of the most distinguished practitioners; and who, if I am correctly informed, were never able to boast of a Medical Diploma. And I might again ask, who has contributed more to raise the profession from a mere mechanical art, to the dignity of a science, and to respectability, than the gentlemen, whose names I have just mentioned ? I answer—none. Then let no arrogant upstart, whose only pretension to a knowledge of dental science, is, a Medical Diploma; say, that none except graduates in medicine are competent to discharge the du-

---

NOTE. I take the liberty of adding to this catalogue the name of C. S. BREWSTER, who is extensively known on the continent of Europe, as the *American Dentist* in Paris. Mr. Brewster has been in the French Capital about six years, and has effected a complete revolution in the modes of practice in that city, having placed himself decidedly at the head of his profession in continental Europe, and secured the confidence and patronage of the royal family of France, and of more of the nobility, as I am credibly informed, than any other man there. All this has been effected within the compass of a few years, by a man who lays no claim to the title of M. D.; and of whom, therefore, it can never be truly said, that a diploma is his only evidence of a liberal education, and of correct professional knowledge.

Mr. Brewster wrote to the author of this note a few days since, acknowledging the receipt of the first number of this Journal, the objects of which he commends in the highest terms of praise, and, in the most friendly manner, proffers his hearty co-operation in its support.

How unlike this gentlemanly conduct in relation to our unremunerated efforts to disseminate useful information to the profession, was the language and deportment of Doct. J. W. Crane, an M. D. Dentist of this city, who stated to the agent when he called on the members of the profession for the purpose of obtaining subscriptions, as the agent informed us, that he should not subscribe for the Journal of Dental Science, because no one of the individuals in New-York, who have the superintendence of its publication, is an M. D., adding, that no one who had not received a Medical Diploma, could be a Dentist.

I would respectfully request some of our mathematical friends to solve the following equation in what was denominated, in my school-boy days, the golden rule of Three:—If one such man as the former *without the title of M. D.*, has, by his talents, knowledge and liberal views, been able to instruct the Dentists, and to elevate the character of the Dental profession in one of the greatest Kingdoms of Europe, how many such men as the latter, *with the title*, would be required to instruct the Dentists, and give dignity to the same profession in a single city of America ?

I feel happy in saying that all of the first Dentists in our country, from whom we have been able to get returns, as well those who have been educated to the medical profession, as those who have not, (and we have many of both descriptions who would do honor to any profession,) have been friendly to our efforts, and have liberally subscribed in order to aid us in our enterprize, undertaken not only for the good of our own profession, but for the benefit of the public at large.

NEW-YORK EDITOR.

ties of the art. There are many dental practitioners, who, although they are not graduates in medicine, are much better read, in many, if not all of its branches, than many who have graduated in them.

Having previously enumerated the branches of knowledge that should be understood by a dental practitioner, nothing which has been here said can be understood as opposing the claims of medical gentlemen, to skill in this particular department of the healing art, on the contrary, I would advise all intending to embrace it, to make themselves thoroughly acquainted with the pathology of disease generally, with therapeutics, and with anatomy, physiology and surgery ; and if possible, with all the branches of medical and surgical science.—My remarks are intended to apply only to a few, who, presuming on their Medical Diplomas as furnishing all the information necessary to be possessed on the subject, scruple not to detract from the well-earned reputation of those who are unable to boast of such honor. Whenever, therefore, I hear a person say, that none except graduates in medicine are competent to discharge the duties of a practitioner of dental surgery, I at once take it for granted, that he himself, is either ignorant of the profession, or wilfully intends to deceive, and in which case, I can regard him in no other light, than as an impostor and empiric.

The studentship in dentistry, that is usually required, is too short for the obtaining of any thing more than a very superficial idea of a few of its general principles. I have heard of individuals commencing its practice in four, five or six, and in some instances, in one week after commencing its study, and that too, without reading a single book upon the subject, or so much as even touching an instrument. In fact, many commence its practice without any instruction at all. I have often heard of persons employed in the laboratories of dentists, where they have no opportunity of acquiring any information on the subject, except what relates merely to the preparation of artificial teeth, setting themselves up as practitioners of the art in all its various branches. An instance of this kind, came under my own personal observation some two or three years since. It was the person of a young man—a jeweller, whom I had had in my employ for a few months for the purpose above mentioned. As soon as he had become tolerably expert in the soldering and finishing of artificial teeth, he imagined himself sufficiently informed on every subject pertaining to the practice, to engage in it himself. He accordingly left me, and went to the south, where he advertised himself as a dentist. Some have engaged in its practice without any other information than that which they acquire from the manufacture of dental instruments.

But it is not to be supposed, from what I have here said, that I regard any previous occupation, as disqualifying a person for the profession ; so far from that, I do not hesitate to say, that I have known individuals, from the avocations just referred to, to rise to the highest point of excellence in it. What I mean to say is, that no man is competent to practice dental surgery, without first being properly instructed in it. As well might a man attempt the practice of law, or medicine without any preparatory study, as that of dentistry ; and the time required for the acquisition of a knowledge of either of the former, is not, perhaps more than is necessary for a thorough instruction in the latter.

How then can we expect a person, ignorant alike of theory and practice, to exercise the duties of a profession, requiring qualifications of the most peculiar kind,—qualifications which can only be attained by a pupilage of from eighteen months to three years, under an individual thoroughly versed in all its branches ? And, while this is permitted, is it to be wondered that there should be empirics in it, or that it should be exposed to reproach ? Strange indeed, would it be, for a person to possess himself of a knowledge of it, without study or instruction, and practice it with judgment and skill; and still more strange would it be, were those, who suffer from its abuses, to possess an unimpaired confidence in its utility. To suppose the first, is worse than absurd; and the last, implies a credulity greater than the history of the past authorizes us to believe.

As long, therefore, as dental surgery is permitted to be practiced without any regard to the qualifications of its practitioners, we need not expect a general confidence in its utility; much less, a proper respect for those engaged in the exercise of its duties. That it has attained the respectability and character for usefulness which it has,—that it has endeared itself to suffering humanity, by the relief which it often affords to the most torturing pain, and the very health it frequently brings to those suffering under languishing disease, is to be attributed to those of its practitioners, who, actuated by a high sense of honor, a noble and praiseworthy ambition for emulation, have devoted themselves to it with the most ardent zeal, and scrupulous integrity—giving up to the increase of the sphere of its usefulness, all their powers and energies of mind, have practiced it only in its greatest perfection. To this class of practitioners—and there is not, perhaps any country in the world which can boast more than this—the meed of too much praise can never be awarded.

Returning however to the point more immediately at issue,—I would remark, that although some contend that a knowledge of none of the branches of medical or surgical science, is essential to a dental practitioner, the better informed of the profession concur in opinion, that in order to the judicious and skilful treatment of many of the diseases that properly belong to this department of the healing art, several of them, at least, should be well understood. Indeed, that a contrary opinion should exist, can only be accounted for, by attributing to those who hold it a most woful ignorance on the subject.

In the treatment of diseases of the mouth, as well as those of other parts of the body, reference should be had to the causes that produce them, and it should be borne in mind, that they are not always idiopathic. They are sometimes symptomatic of some other disease, and when this is the case, require other remedies than local; and, how are they to be prescribed by a person ignorant alike of pathology and therapeutics. The curative indications of the same disease often vary, and consequently, can only be correctly followed out by a person acquainted with the several parts of the physical structure—the respective relationships which they bear to each other,—the doctrine of their various morbid conditions and associations, and the *modus operandi* of the remedies that have been furnished us. For example, odontalgia is sometimes the result of gestation; at other times, of a disordered state of the stomach or digestive apparatus; and diseased gums, although principally dependent on local irritation, are frequently favored by a cachectic tendency of the general system. An impaired or vitiated state of the juices of the mouth—a most prolific source of disease, not only to the teeth and gums, but also to the alveolar processes, is often caused by an unhealthy diathesis in some other part of the body. Thus it will be perceived, that in the treatment of many of the maladies of the mouth, constitutional remedies will be required; and although the dentist may say, with other diseases he has nothing to do, it is at least requisite for him to understand them, in order to detect the agency they may have in the production of those, the treatment of which, does properly belong to gentlemen of the dental profession.

Nor is a knowledge of these branches any less necessary to enable the dental practitioner to detect the agency, which a morbid condition of the teeth, gums and alveoli exert in the production of disease in other parts, than for that of those in other parts, in the generating of disease in these; and that they do this, is no longer a matter of conjecture. Instances of the deleterious effects of the diseases of these parts, on the functional operations of other organs, and the system generally, are occurring daily; and Dr. S. Brown, when he says, in Dentologia—

"So, when disease invades the dental arch,
"And strides in anguish on his angry march,
"His burning touch, like the electric flame,
"Flashes through every fibre of the frame,"

exaggerates the fact but little, if he does any at all. The real amount of disease that is produced in this way, could it be known, would present a most startling picture; and here permit me to observe, that with how much soever of truth may be charged upon many of the members of our profession a want of sufficient attention to the influences exerted upon the teeth, gums, &c. by the diseases of the general system, many of the medical, are no less remiss in regard to those of the morbid conditions of these parts, upon the functional operations of other parts of the body. By attending to them, the success of the physician would be as much increased, as would that of the dentist by attending to the others.

It is not sufficient that the dentist knows how to extract and to plug a tooth, and even to supply the loss of these organs with artificial ones; he should be acquainted with all of their morbid associations as well as those of their contiguous parts, if he would be as useful as his profession is capable of making him.

The proposition, that anatomy, physiology, pathology and therapeutics are necessary to a thorough education in dentistry, being established, the next point to be considered is, in what way a knowledge of the general principles of surgery is to be beneficial to a practitioner in this particular department of the profession.

In the determining of this question, it may be necessary, in the first place, to define what surgery is. To do this, or to tell wherein it differs from physic, is no easy task, since scarcely any two can be found to agree as to the proper definition. So intimately, and I might say, inseparably are the two connected that it would be difficult to determine the line that separates the one from the other. In fact, the province of the physician and surgeon encroach so much upon each other, that it would be difficult to exercise the duties of one without trespassing upon the other; so that in reality, every physician should be a surgeon, and every surgeon a physician. Surgery is defined by some as that branch of medical science, which has for its object, the cure of disease by the use of instruments, or local applications. This however, is but an imperfect definition of the term; its signification is by far more extensive, for there is a wide difference between a mere operation, and the subsequent treatment of the constitutional symptoms that supervene. Surgery means something more than the amputation of an arm or a leg, the reduction of a fracture or dislocation, the excission of a tumour, the dressing of a sore, or the application of a bandage. It would seem rather to mean that union of medical science which embraces a knowledge of all the parts of the human organization separately and as a whole—the functions of all its organs individually and reciprocally—the character of the effects produced upon it by influences of every description, both local and general—and the manual arts, whereby its various local affections, whether external or internal that arise in consequence of these influences, are cured by the employment of instruments, and such medical treatment, local and general, as may be called for by supervening symptoms. Thus it is seen, that surgery, instead of being altogether a manual occupation, is a *union of three sciences;* namely, that of *anatomy, disease, and so much of mechanics, as, in connexion with these, is necessary for the cure of certain local and constitutional affections of the human organization.*

Although it will be seen from the foregoing explanation, that surgery embraces all the other branches of medical science that have been noticed as being essential to the practitioner, yet as the duties of the physician and surgeon, have, and perhaps, for the greater perfection of their exercise, very properly assumed to a certain extent distinctive characters, it was deemed advisable, in presenting their claims to the attention of the profession, to speak of them separately.

Regarding surgery in the light in which it has here been presented, can any one question the importance of a knowledge of its general principles, to the dental practitioner? That it is important, and highly so too, must, I think, be apparent to every one conversant with the duties that properly devolve on, and which are now confided to those engaged in this branch of the practice. Were the duties of the dentist confined exclusively to the treatment of the diseases of the teeth alone, it might perhaps be dispensed with, or at any rate, would not be so essential; but when it is recollected, that the diseases of these organs, however simple they may seem, abstractly considered, are by the relationships which they have with the organism generally, and to the parts with which they are more immediately connected, often exceedingly complicated; and that it is the province also of the dental practitioner, to take charge of the gums and alveolar processes, and that some of these are by far more complicated and difficult of cure than any that attack the teeth, requiring in their management, both local and constitutional remedies, the importance of surgical knowledge in this department admits not of doubt.

The treatment of most of the diseases of the gums and alveolar processes, and especially those requiring manual operations, was formerly confided to the regular surgeon; but latterly, for the most part, they are entrusted to the dentist, whose better opportunity for studying their peculiarities enables him to manage them, if not with more skill, at least, with greater facility and convenience; and if he would make himself as useful in every department of his vocation as he should be, he should avail himself of all the lights that are to be had on the subject: and from the medical and surgical sciences he will be able to derive many that can be obtained from no other source.

After having informed himself sufficiently on these branches, his attention should next be directed to the special study of the diseases of the teeth, gums, alveolar processes and antrum maxillare. With these, and their treatment, he should make himself thoroughly acquainted. This done, his next care will be, the acquisition of a knowledge of the mechanical parts of the profession, and for which he should serve an apprenticeship under an experienced practitioner, long enough to enable him to execute with skill, all kinds of dental mechanism.

By this, I mean not only the preparation of artificial teeth for insertion, but also for the arranging, antagonizing, and applying of them to the mouth. There are many that can prepare artificial teeth to a model, who know nothing of the manner of arranging, antagonizing and applying them ; and if these be not properly attended to, they will be of little, and frequently of no use at all. The skill of the dentist, therefore, is displayed as much and even more, in determining the manner of preparing artificial teeth, than it is in preparing them, for upon the correctness of his judgment in this, their utility will in a great measure depend.

Having thus briefly glanced at the branches of knowledge that should be comprised in the professional education of a dentist, and at some of the consequences that too frequently result from the practice of those who do not sufficiently understand them ; it may be well, before proceeding to the enquiry, how the evils of which mention has been here made, are to be remedied, to remark, that however indispensable theoretical information is to skill in dentistry, it is not all that is requisite. To excel in the profession, a man should give to it his whole attention. In fact, this is necessary in any thing, but more especially in this, than almost any other avocation. This is no new doctrine ; it obtained at a very remote period, and so fully were the ancient Egyptians impressed with the belief that a man could not be skilled in more than one occupation, that they restricted their physicians to the treatment of a single disease. Every disease, therefore, in those times had its physician, and had he not been compelled by law to follow certain fixed rules, he would doubtless have attained to very great perfection.

But with this part of the subject I have done. Enough has been said to show the importance of a more extensive and thorough education in dentistry, than is possessed by a vast many of its practitioners, and that to a deficiency in this, most of the abuses of the art are to be attributed. Let those who practice dentistry be well educated in it, and my word for it, the profession will escape the reproaches that have been but too frequently heaped upon it, and will rise in respectability to a level with those of the most favored and useful. But how the condition of the profession is to be bettered, is a question perhaps somewhat difficult of solution, since, as Dr. Bell, editor of the "Eclectic Journal of Medicine" in a notice of the "American Journal of Dental Science" very truly remarks, "the calling of a dentist seems to be considered open ground, into which any fellow who has impudence, some steadiness of hand, and a case of instruments, thinks himself free to take up a position."

Of the impositions in dentistry, the profession are fully conscious; hardly a day passes that examples of the most palpable mal-practice do not come to the observation of every practitioner; and with a knowledge of these facts, should we rest satisfied without making an effort to prevent their future occurrence? Do we not owe this to our own reputation, and do we not, more especially, owe it to those from whom we derive our employment? To these questions there can be but one answer,— We do! Then let us bestir ourselves, and unite in the adoption of such measures as shall tend to the accomplishment of an object so much to be desired; and which, should they be successful, will reflect upon us the highest credit.

Were I permitted to propose a plan for the remedying of the evils here complained of, it would be, to petition the legislatures of the states, within which we respectively reside, to pass laws prohibiting the practice except by persons who should be regularly authorized to do so, and the authority to emanate from persons competent to judge of the capabilities that should be possessed by those who exercise the duties of the profession. Were the enlightened of the profession to unite in a measure of this kind, their petitions would be granted without a doubt, for community at large have experienced too much of the bad effects growing out of the ignorance of dental practitioners.

But we should not stop here,—we should furnish the necessary facilities for those who may design practicing the art, to qualify themselves properly for its duties. Dentistry should be as much a subject of public instruction, as medicine or surgery; and to me it has been a matter of much surprise, that the efforts of the better informed of the profession, have not been directed to the establishment of an institution for this purpose. An institution of this kind, would not only redound to the credit of the whole profession, but it would be instrumental of much general good. Its influence could not be otherwise than salutary, as it would have a tendency to drive from the ranks of the profession ignorant pretenders, and substitute in their place, men that would be qualified to practice with credit to themselves and to the benefit of their patients. Men of genius and education would be more frequently induced to enter it, for then none except competent persons would be recognized as its members.

That a college for the education of persons for the profession might be gotten up, and that it would be well sustained, I think is more than probable, for if a school of this kind was in operation, it would be expected of dental practitioners, that they should be educated in it, or at least, those who should subsequently enter the profession.

The same object might be accomplished by the establishing of professorships of dentistry in the Medical Schools. All the branches necessary to a dental education, might be taught; and should the profession not be disposed to establish a school for their own exclusive benefit, it is to be hoped the importance of the subject may be soon so felt, as to induce the medical institutions of the country to take it up, and furnish the necessary facilities for the obtaining of as thorough an education in this, as in their own immediate profession.*

* The late, much lamented Edward Hudson of Philadelphia, received, some years previous to his death, from the Medical College of that city, the honorary title of Dr. of Medicine; and H. H. Hayden of Baltimore, received in the same way, the same honorable distinction from the College of Maryland, and also from the College of Philadelphia. Similar honors were never awarded to any individuals who more truly deserved them. They were given, not for what titles would enable them to perform for the profession of dentistry, but for what their talents, industry, skill and stern integrity had already done for it.

I most earnestly advise all young gentlemen who are qualifying themselves for our profession, to become thoroughly versed in all those branches of science so ably recommended in the foregoing article. That no dentist can practice successfully in all cases without such knowledge, I readily admit ; but I have yet to discover that the granting of a diploma by a chartered medical institution, or the purchasing of one from some of the medical schools, or from individuals for a few dollars, as is frequently the case, will impart this knowledge.

The same discovery remains yet to be made in England, for I have not yet known among all those who have been distinguished there, either as practitioners or writers, one who has thought it worth his while to append to his name the title of M. D., not even the celebrated John Hunter—nor at the present day, the highly distinguished Sir Samuel Cartwright, nor the accurate and profound writer, Thomas Bell. N. Y. ED.

